# No role for quality scores in systematic reviews of diagnostic accuracy studies

**DOI:** 10.1186/1471-2288-5-19

**Published:** 2005-05-26

**Authors:** Penny Whiting, Roger Harbord, Jos Kleijnen

**Affiliations:** 1 MRC Health Services Research Collaboration, Department of Social Medicine, University of Bristol, Bristol, UK; 2 Centre for Reviews and Dissemination, University of York, York, UK

## Abstract

**Background:**

There is a lack of consensus regarding the use of quality scores in diagnostic systematic reviews. The objective of this study was to use different methods of weighting items included in a quality assessment tool for diagnostic accuracy studies (QUADAS) to produce an overall quality score, and to examine the effects of incorporating these into a systematic review.

**Methods:**

We developed five schemes for weighting QUADAS to produce quality scores. We used three methods to investigate the effects of quality scores on test performance. We used a set of 28 studies that assessed the accuracy of ultrasound for the diagnosis of vesico-ureteral reflux in children.

**Results:**

The different methods of weighting individual items from the same quality assessment tool produced different quality scores. The different scoring schemes ranked different studies in different orders; this was especially evident for the intermediate quality studies. Comparing the results of studies stratified as "high" and "low" quality based on quality scores resulted in different conclusions regarding the effects of quality on estimates of diagnostic accuracy depending on the method used to produce the quality score. A similar effect was observed when quality scores were included in meta-regression analysis as continuous variables, although the differences were less apparent.

**Conclusion:**

Quality scores should not be incorporated into diagnostic systematic reviews. Incorporation of the results of the quality assessment into the systematic review should involve investigation of the association of individual quality items with estimates of diagnostic accuracy, rather than using a combined quality score.

## Background

Quality assessment is as important in systematic reviews of diagnostic accuracy studies as it is for any other systematic review. One method of incorporating quality into a review is to use a quality score. Quality scores combine the individual items from a quality assessment tool to provide an overall single score. One of the main problems with quality scores is determining how to weight each item to provide an overall quality score. There is no objective way of doing this and different methods are likely to produce different scores that may lead to different results if these scores are used in the analysis.

There has been much discussion regarding the use of quality scores in the area of clinical trials[1-8]. Although this discussion has not been specific to diagnostic accuracy studies much of these discussions also apply to this topic area. Previous work illustrating the problems associated with quality scores has used different scales, which not only weighted items differently but also included different items[9]. It has been argued that it was the differences in the items covered by the tools that contributed to the differences found, rather than the use of a combined quality score[2,3,6]. The debate regarding quality scores remains and quality scores continue to be used as part of the quality assessment process in both therapeutic and diagnostic systematic reviews [10-14]. The Jadad scale, one of the most commonly used quality assessment tools for therapeutic studies, incorporates a quality score[15], as does one of the commonly used diagnostic quality assessment tools[16]. A recent review of existing quality assessment tools for diagnostic accuracy studies found that 12 of 67 tools (18%) incorporated a quality score[17]. A further review of how quality assessment has been incorporated into systematic reviews found that 16% of reviews that performed some form of quality assessment used quality scores as part of this assessment[18].

We are not aware of any work that has looked at the effect of using different weightings for the same quality assessment tool to produce an overall quality score or that has been done in the area of diagnostic accuracy studies. This project presents a practical example of the problems associated with the use of quality scores in systematic reviews. The aim is to use QUADAS, a quality assessment tool that we recently developed to assess the quality of diagnostic accuracy studies included in systematic reviews[19], to investigate the effect of different weightings on estimates of test performance.

## Methods

### Scoring methods

QUADAS does not incorporate a quality score. We therefore developed five different schemes for weighting QUADAS (Table 1) to produce an overall study quality score:

**Table 1:** QUADAS and scoring guide for each of the 5 schemes

**QUADAS Item**		**Scoring scheme**				
		
		**1**	**2**	**3**	**4**	**5**
1	Was the spectrum of patients representative of the patients who will receive the test in practice?	1	2	2	3	10
2	Were selection criteria clearly described?	1	2	1	1	2
3	Is the reference standard likely to correctly classify the target condition?	1	2	3	2	10
4	Is the time period between reference standard and index test short enough to be reasonably sure that the target condition did not change between the two tests?	1	2	3	1	6
5	Did the whole sample or a random selection of the sample, receive verification using a reference standard of diagnosis?	1	2	3	3	9
6	Did patients receive the same reference standard regardless of the index test result?	1	2	3	2	7
7	Was the reference standard independent of the index test (i.e. the index test did not form part of the reference standard)?	1	2	3	1	7
8	Was the execution of the index test described in sufficient detail to permit replication of the test?	1	2	2	1	3
9	Was the execution of the reference standard described in sufficient detail to permit its replication?	1	2	2	1	2
10	Were the index test results interpreted without knowledge of the results of the reference standard?	1	2	3	3	8
11	Were the reference standard results interpreted without knowledge of the results of the index test?	1	2	3	3	6
12	Were the same clinical data available when test results were interpreted as would be available when the test is used in practice?	1	2	3	3	5
13	Were uninterpretable/ intermediate test results reported?	1	2	1	1	4
14	Were withdrawals from the study explained?	1	2	1	1	3

**Total score**		**14**	**28**	**33**	**26**	**85**

All scoring given above refer to the score which studies that answered "yes" to each question should be given. Studies that answered "no" or "unclear" were scored 0 for each scoring system with the exception of system 2 in which studies that scored "unclear" were given 1.

#### 1. Equal weighting

All items were weighted equally and scored 1 for yes and 0 for no or unclear.

#### 2. Equal weighting accounting for unclear

All items were weighted equally but scored 2 for yes, 1 for unclear and 0 for no.

#### 3. Weighting according to item type

Items which aimed to detect the presence of bias were scored 3 for yes (items 3, 4, 5, 6, 7, 10, 11, 12), items which aimed to detect sources of variation between studies were scored 2 for yes (item 1) and items which were related to the quality of reporting were scored 1 for yes (items 2, 8, 9, 13, 14). All items were scored 0 for no or unclear.

#### 4. Weighting based on the evidence

The evidence used in the development of QUADAS was used to determine item weighting[18]. Two systematic reviews of the diagnostic literature provided an evidence base for the development of QUADAS. The first was a review of evidence on factors that can lead to bias or variation in the results of diagnostic accuracy studies[20]. For each source of bias or variation, the number of studies that found that a particular source of bias or variation impacted on estimates of diagnostic accuracy was summarised. The second review considered all existing quality assessment tools designed for diagnostic accuracy studies[17]. The proportion of tools that covered each of a list of possible items relating to the quality of diagnostic accuracy studies was summarised. To estimate quality scores using this weighting scheme, items for which there was evidence of bias or variation from at least 5 studies or which were included in at least 75% of existing quality assessment tools were scored 3 for yes (items 1, 5, 10, 11, 12); items for which there was evidence of bias from at least 2 studies and which were included in at least 50% of existing quality assessment tools were scored 2 points for yes (items 3, 6). All other items were given 1 point for yes (items: 4, 7, 8, 9, 13, 14). All items were scored 0 for no or unclear.

#### 5. Subjective scoring

Each item was scored from 1 – 10 based on one of the author's subjective opinion of its importance. This allowed items which the author considered to be of greater importance to receive a much greater weighting than items considered less important. For example items such as inclusion of an appropriate patient spectrum and the use of an appropriate reference standard were judged to be much more important than items such as reporting of selection criteria or details of the reference standard. This is reflected in the weightings given to these items.

These weighting schemes are summarised in Table 1. Each different weighting scheme was used to produce an overall quality score, giving a total of five different scores for each study. As the total maximum possible points differed across the scoring schemes, the scores were expressed as the percentage of the maximum possible points for each scoring scheme so that the quality scores could be compared across schemes.

### Data set

We selected a data set consisting of 28 studies that looked at ultrasound for the diagnosis of vesico-ureteral reflux in children. These came from a systematic review on the diagnosis and further investigation of urinary tract infection (UTI) in children under 5[21]. The studies were selected as they provided a set of studies that were heterogeneous in terms of quality and individual study results. They provide two separate data sets within one larger data set as they can be split according to the type of ultrasound used: contrast-enhanced (16 studies) or standard ultrasound (12 studies). Although both types of study evaluated ultrasound and so involve similar quality issues, there were differences in accuracy between the ultrasound types: contrast-enhanced ultrasound is a much more accurate test for vesico-ureteral reflux in children than standard-ultrasound.

Thus we were able to investigate whether different quality scores have the same impact on two separate data sets. QUADAS was used in this review to assess the quality of studies. All studies had previously been coded using QUADAS as yes, no or unclear. This coding was carried out by one reviewer and checked by a second reviewer.

### Analysis

Methods for investigating the effects of the quality scores on test performance We used three different methods to investigate the effects of quality scores on test performance. Each method was performed separately for the standard ultrasound studies and for the contrast-enhanced ultrasound studies. For each of the steps involving pooling of studies, standard SROC (summary receiver operating characteristic) methods were used to pool individual study results[22]. The SROC model was estimated by regressing D (log(DOR), where DOR is the diagnostic odds ratio) against S (logit (sensitivity) + logit (1-specificity)), weighting according to sample size, for each study. To account for zero cells in the 2 × 2 tables, 0.5 was added to every cell for all 2 × 2 tables as recommended by Moses et al.[22]. All analyses were carried out using STATA version 8 (StataCorp, College Station, Texas).

#### a. Ranking of studies

Studies were ranked according to quality score and we investigated whether the ranking of each study was different according to the method used to weight the quality scores. This allowed investigation of whether the use of a summary quality score in a table as an overall indicator of quality is appropriate.

#### b. Difference in estimates diagnostic accuracy between high and low quality studies

We stratified studies into "high" and "low" quality studies using the quality score. The median quality score was calculated for each scoring scheme. Studies with scores higher than the median score were classified as "high" quality studies, while studies with the median quality score or lower were classified as "low" quality studies. A relative diagnostic odds ratio (RDOR) was calculated for each of the different quality scores by dividing the pooled diagnostic odds ratio (DOR) for the high quality studies by that for the low quality studies.

#### c. Quality score as a possible source of heterogeneity

The effects of quality on test performance were investigated using meta-regression analysis. The SROC model was extended to include "quality score" as a continuous variable, assuming a linear relationship between quality score and log DOR. We calculated the RDOR for a 10 point increase in quality by multiplying the coefficient for the quality score obtained from the regression analysis by 10 and then anti-logging it.

## Results

**Figure 1:**
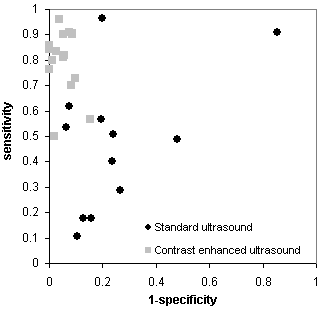
Estimates of sensitivity and 1-specificity plotted in ROC space for standard and contrast enhanced ultrasound

Table 2 summarises the results for the 28 studies included in this study. It presents the 2 × 2 table results for each study, the results of the quality assessment, and the summary quality scores produced using each of the five scoring schemes. Reading table 2 vertically per item allows readers to make some judgments about which items might contribute to variations in the scores. Figure 1 shows the results of the studies plotted in receiver operating characteristic (ROC) space, giving an indication of the heterogeneity between studies.

**Table 2:** Individual study results (2 × 2 data), results of the quality assessment, and quality scores using each of the five scoring schemes

**Study details**	**2 × 2 Data**				**QUADAS Results**														**Quality score**				
	
	**TP**	**FP**	**FN**	**TN**	**Spectrum composition**	**Selection criteria**	**Reference standard**	**Disease progression bias**	**Partial verification bias**	**Differential verification bias**	**Incorporation bias**	**Test execution details**	**Reference execution details**	**Test review bias**	**Diagnostic review bias**	**Clinical review bias**	**Uninterpretable results**	**Withdrawals**	**Score 1**	**Score 2**	**Score 3**	**Score 4**	**Score 5**
**Standard US**																							

Baronciani (1986) [24]	13	4	8	49	+	+	+	?	+	+	+	-	-	?	?	?	?	?	43	64	45	46	53
Dura (1997) [25]	3	4	14	27	-	+	+	+	+	+	+	+	+	+	+	?	+	+	86	89	85	77	79
Evans (1999) [26]	2	10	17	84	-	+	+	?	+	+	+	+	-	?	?	?	+	?	50	68	48	42	49
Foresman (2001) [27]	24	43	25	47	-	+	+	+	+	+	+	+	-	+	+	?	+	+	79	82	79	73	76
Mage (1989) [28]	22	5	19	76	-	-	+	?	+	+	+	+	-	?	?	?	?	?	36	57	42	35	42
Mahant (2002) [29]	14	30	21	97	-	+	+	+	+	+	+	+	-	+	+	?	?	?	64	75	73	65	68
Morin (1999) [30]	20	41	2	7	-	+	+	+	+	+	+	+	-	?	?	?	?	?	50	68	55	42	52
Muensterer (2002) [31]	35	76	34	241	-	+	+	+	+	+	+	+	-	?	?	?	?	+	57	71	58	46	55
Oostenbrink (2000) [32]	21	20	16	83	+	+	+	?	-	?	+	+	-	+	?	?	?	-	43	61	42	42	47
Salih (1994) [33]	26	3	1	12	+	-	+	+	+	+	+	-	-	?	?	?	?	-	43	57	52	46	58
Tan (1988) [34]	3	6	14	32	-	-	+	+	+	+	+	+	+	-	-	?	?	?	50	61	58	42	52
Verber (1988) [35]	8	9	20	25	+	-	+	?	-	+	+	+	+	?	?	?	?	-	43	61	45	38	46

**Median Score**																			**50**	**66**	**53**	**44**	**52**

**Contrast-enhanced US**																							

Alzen (1994) [36]	20	6	2	73	-	-	+	+	+	+	+	-	-	?	?	?	?	?	36	54	45	35	46
Bergius (1990) [23]	56	2	14	176	+	-	+	?	+	+	+	+	-	+	+	+	?	-	64	71	76	81	76
Berrocal (2001) [37]	94	29	10	307	-	-	+	+	+	+	+	+	-	?	?	?	?	?	43	61	52	38	49
Berrocal Frutos (2000) [38]	63	19	7	204	-	+	+	+	+	+	+	+	+	+	+	?	+	+	86	89	85	77	79
Haberlick (1997) [39]	21	10	9	114	-	+	+	+	+	+	+	+	-	?	?	?	?	?	50	68	55	42	52
Kessler (1982) [40]	13	0	4	38	-	+	+	?	-	+	+	+	-	+	?	?	?	-	43	57	45	38	44
McEwing (2002) [41]	8	3	8	173	-	+	+	+	+	+	+	+	-	+	+	?	?	+	71	79	76	69	72
Mentzel (2002) [42]	36	10	4	174	-	-	+	+	+	+	+	+	+	+	?	?	+	+	71	79	73	62	69
Nakamura (2002) [43]	9	3	2	52	-	-	+	?	+	+	+	+	-	+	+	?	?	?	50	64	61	58	59
Piaggio (2003) [44]	42	35	32	196	-	+	+	?	+	+	+	+	+	?	?	+	?	+	64	79	64	58	56
Radmayr (2002) [44]	71	5	3	129	-	-	+	+	+	+	+	+	+	+	+	?	+	+	79	82	82	73	76
Schneider (1984) [45]	46	15	17	141	-	+	+	+	+	+	+	+	+	+	+	?	?	?	71	82	79	69	71
Siamplis (1996) [46]	15	4	3	154	-	+	+	+	+	+	+	+	-	?	?	?	+	?	57	71	58	46	56
Valentini (2001) [47]	34	4	8	72	-	-	+	+	+	+	+	+	+	-	-	?	?	?	50	61	58	42	52
Uhl (2003) [48]	16	0	3	28	+	+	+	+	+	+	+	-	-	+	?	?	+	?	64	75	67	65	74
Von Rohden (1995) [49]	6	0	1	19	-	+	+	+	+	+	+	+	+	+	+	?	+	+	86	89	85	77	79

**Median score**																			**64**	**73**	**65**	**60**	**64**

TP = true positives; FP = false positives; FN = false negatives; TN = true negatives + = yes; - = no; ? = unclear

### a. Ranking of studies

**Figure 2:**
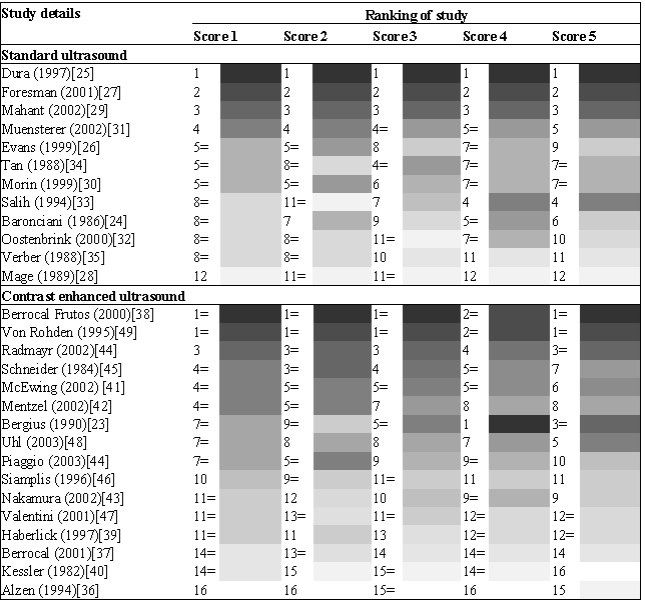
ranking of studies according to each different quality score

The ranking of the studies using the different quality scores is summarised in Figure 2. For standard ultrasound, all scoring schemes ranked the same three studies as being the best studies, and ranked these in the same order. All scoring schemes also ranked the same study as being of the worst quality. For contrast enhanced ultrasound, scores 1, 2, 3 and 5 ranked the same two studies as being of the best quality. Score 4 ranked these two studies as having the second highest quality score. The study ranked as being the best quality study by score 4 was ranked as being of intermediate quality by the other scoring schemes. All scores ranked the same three studies as being of worst quality, with scores 1, 2, 3 and 4 ranking them in the same order. For both types of ultrasound the different scoring schemes ranked the more intermediate quality studies in different orders.

### b. Difference in estimates of diagnostic accuracy between high and low quality studies

**Figure 3:**
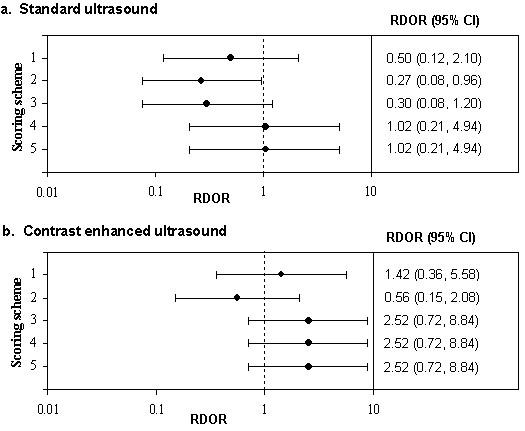
Forest plots showing the RDOR in "high" quality studies compared to "low" quality studies for each of the five quality scoring schemes

The RDOR comparing studies classified as "high" to those classified as "low" quality using each of the five scoring schemes is shown in Figure 3, separately for standard ultrasound and contrast enhanced ultrasound. For standard ultrasound, scores 1,2, and 3 gave RDORs suggesting that high quality studies produced lower estimates of diagnostic accuracy than low quality studies. In contrast, the results from schemes 4 and 5 suggested that there was no difference in estimates of the DOR between high and low quality studies. For contrast-enhanced ultrasound, scores 1, 3, 4 and 5 all classified the same set of studies as being of high and low quality. The RDORs for these quality scores suggested that high quality studies produce higher DORs than low quality studies. In contrast, scheme 2 produced an RDOR suggesting that high quality studies produce lower estimates of diagnostic accuracy than low quality studies.

### c. Quality score as a possible source of heterogeneity

**Figure 4:**
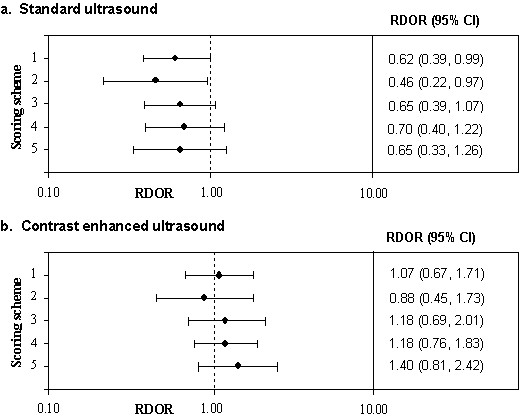
Forest plots showing the RDOR for a 10 point increase in quality for each of the 5 quality scoring schemes

Figure 4 shows the RDORs for a 10 point increase in quality score for each of the five different quality scores, separately for standard and contrast-enhanced ultrasound. For standard ultrasound, all scoring schemes suggested that high quality studies produce lower DORs than low quality studies. For contrast-enhanced ultrasound, scores 1, 3, 4 and 5 suggested that higher quality studies produce higher DORs than lower quality studies, while score 2 suggested that they produced lower estimates. However, the confidence intervals around these estimates were wide and all included one.

## Discussion

This study has shown that using different methods of weighting individual items from the same quality assessment tool can produce different quality scores. Incorporating these quality scores into the results of a review can lead to different conclusions regarding the effect of study quality on estimates of diagnostic accuracy.

Although the ordering of studies using the different quality scores were broadly similar, there were some differences which could lead to different conclusions if they were used in a systematic review. For example, for the contrast enhanced ultrasound studies, if quality scoring scheme 4 or 5 was used then the study by Bergius and colleagues[23] would be considered to be one of the best quality studies. However, if scoring schemes 1, 2, or 3 were used then this study would be considered to be an average quality study. This suggests that quality scores should not be used as a summary indicator of quality in results tables in systematic reviews. Instead either the results of the whole quality assessment, or key components of the quality assessment, should be reported.

Stratifying studies into high and low quality studies according to quality score also varied according to the scoring scheme used. Although the confidence intervals for all comparisons were wide and all but one included one, the conclusions regarding the association of study quality and diagnostic accuracy differ according to the scoring scheme used. It is important to note that in practice a reviewer would only use one scoring scheme and so the results from the other scoring schemes would not be available to them: they would have to draw conclusions from the results for the single scoring scheme that they selected. For standard ultrasound, two of the schemes assessed produced an overall quality score that suggested no association between study quality and the diagnostic odds ratio. However, if the other three schemes were used then the conclusion would have been that high quality studies tend to produce lower estimates of diagnostic accuracy than low quality studies. Similarly for contrast-enhanced ultrasound, the conclusion for four of the scoring schemes was that high quality studies tend to produce higher estimates of diagnostic accuracy than low quality studies. In contrast, if the other scoring scheme had been used the conclusions would have been reversed. These results suggest that the use of quality scores to stratify studies into high and low quality studies should be avoided.

The inclusion of quality score as a continuous variable in the meta-regression showed fewer differences between scoring schemes. There were larger associations between quality score and the DOR for standard ultrasound than for contrast enhanced ultrasound. This would be expected as there was more heterogeneity between studies of standard ultrasound and so there was more variation that could have been explained by differences in quality. For standard ultrasound the direction of the association between study quality and test performance was the same for all scoring schemes. For contrast enhanced ultrasound the associations reported for quality scores were close to one with wide confidence intervals. This suggests very little association between quality score and diagnostic accuracy, although scoring scheme 2 again produced an association in the opposite direction to the other scoring schemes. The investigation of the association of an overall quality score with a summary effect estimate can be complicated. If no association is found between the two, this does not mean that quality does not affect the summary estimate. It may be that there is no association with any of the components of quality incorporated into the score; there may be associations with one or more components but that these have very little weight and are lost in the overall quality score; or it may be that there are association with two or more components but that these act in opposite directions cancelling each other out[7].

It is interesting to note that for the contrast enhanced ultrasound studies that it was generally scoring scheme 2 that produced different results to the other scoring schemes. All other scoring schemes scored studies that answered "unclear" to an item in the same way as studies that answered "no". Scoring scheme 2 scored these studies higher than those that answered "no". The difference between scoring scheme 2 and the other scoring schemes may therefore be related to the quality of reporting of studies: studies that were poorly reported and answered "unclear" to many of the QUADAS items would be rated higher using this scoring scheme than the other schemes.

The results of this study support the finding of Juni and colleagues that using summary scores to identify high quality studies is problematic[9]. We did not find such large differences between the different scoring schemes included in this study as Juni *et al*. This would be expected as we were using different methods of weighting the same quality assessment tool whereas they used different quality assessment tools, each of which not only weighted items differently but also included different items. In addition, we used only five different scoring schemes whereas Juni *et al*. used 25 different quality scales.

Our study was limited by the relatively few primary studies included: for standard ultrasound we included 12 studies, and for contrast-enhanced ultrasound we included 16 studies. The greater the number of studies included in a meta-analysis, the greater the power for detecting associations between study quality and estimates of diagnostic accuracy. If additional primary studies had been available, more precise estimates of the association between quality score and diagnostic accuracy would have been produced and the differences between these associations for the different scoring schemes could have been assessed in more detail. An additional limitation was the poor quality of the reporting of the studies. This resulted in a large proportion of "unclear" responses to the quality assessment.

A further limitation of this study was the lack of a gold standard against which to compare the quality scoring schemes. Lack of agreement between different scoring systems could be expected and does not necessarily invalidate all the scoring systems. The problem in this situation is determining which quality scoring scheme is the most valid. This is an inherent problem with using a quality score, and there is no reliable way of doing this.

## Conclusion

This study, in the area of diagnostic systematic reviews, supports the evidence from previous work in the area of therapeutics suggesting that quality scores should not be incorporated into systematic reviews. Incorporation of the results of the quality assessment into the systematic review should involve a component approach, where the association of individual quality items with test accuracy are investigated individually, rather than using a combined quality score.

## Competing interests

The author(s) declare that they have no competing interests.

## Authors' contributions

Penny whiting contributed to the conception and design of the study, acquisition of data, analysis and interpretation of data, and drafted the manuscript. Roger Harbord and Jos Kleijnen contributed to the analysis and interpretation of data and the critical review of the manuscript for important intellectual content.

## Pre-publication history

The pre-publication history for this paper can be accessed here:


